# Effects of inspiratory flow on lung stress, pendelluft, and ventilation heterogeneity in ARDS: a physiological study

**DOI:** 10.1186/s13054-019-2641-0

**Published:** 2019-11-21

**Authors:** Alessandro Santini, Tommaso Mauri, Francesca Dalla Corte, Elena Spinelli, Antonio Pesenti

**Affiliations:** 1 Department of Anesthesia, Critical Care and Emergency, Fondazione IRCCS Ca’ Granda Ospedale Maggiore Policlinico, University of Milan, Via F. Sforza 35, 20122 Milan, Italy; 2 Deparment of Anesthesia and Critical Care Medicine, Humanitas Clinical and Research Center, Rozzano, MI Italy; 3 Department of Pathophysiology and Transplantation, University of Milan, Milan, Italy; 4 Department of Morphology, Surgery and Experimental Medicine, Azienda Ospedaliera-Universitaria Arcispedale Sant’Anna, University of Ferrara, Ferrara, Italy

**Keywords:** Electrical impedance tomography, Inspiratory flow, ARDS, Heterogeneity

## Abstract

**Background:**

High inspiratory flow might damage the lungs by mechanisms not fully understood yet. We hypothesized that increasing inspiratory flow would increase lung stress, ventilation heterogeneity, and pendelluft in ARDS patients undergoing volume-controlled ventilation with constant tidal volume and that higher PEEP levels would reduce this phenomenon.

**Methods:**

Ten ARDS patients were studied during protective volume-controlled ventilation. Three inspiratory flows (400, 800, and 1200 ml/s) and two PEEP levels (5 and 15 cmH_2_O) were applied in random order to each patient. Airway and esophageal pressures were recorded, end-inspiratory and end-expiratory holds were performed, and ventilation distribution was measured with electrical impedance tomography. Peak and plateau airway and transpulmonary pressures were recorded, together with the airway and transpulmonary pressure corresponding to the first point of zero end-inspiratory flow (P1). Ventilation heterogeneity was measured by the EIT-based global inhomogeneity (GI) index. Pendelluft was measured as the absolute difference between pixel-level inflation measured at plateau pressure minus P1.

**Results:**

Plateau airway and transpulmonary pressure was not affected by inspiratory flow, while P1 increased at increasing inspiratory flow. The difference between P1 and plateau pressure was higher at higher flows at both PEEP levels (*p* < 0.001). While higher PEEP reduced heterogeneity of ventilation, higher inspiratory flow increased GI (*p* = 0.05), irrespective of the PEEP level. Finally, gas volume undergoing pendelluft was larger at higher inspiratory flow (*p* < 0.001), while PEEP had no effect.

**Conclusions:**

The present exploratory analysis suggests that higher inspiratory flow increases additional inspiratory pressure, heterogeneity of ventilation, and pendelluft while PEEP has negligible effects on these flow-dependent phenomena. The clinical significance of these findings needs to be further clarified.

## Introduction

Acute respiratory distress syndrome (ARDS) is a very severe condition, characterized by inflammatory lung edema, hypoxemic respiratory failure, and need for mechanical ventilation [1]. The pathogenesis of ARDS is complex and the outcome is affected by both the underlying disease [2] and the ventilation settings applied [3]. Animal studies using different species showed that too large tidal volumes and/or pressures delivered to the lungs can promote an additional lung injury, indistinguishable from ARDS, named ventilator-induced lung injury (VILI) [4]. Thus, VILI prevention has become a mainstay of treatment of ARDS, changing the objective of mechanical ventilation from obtaining near-normal gas exchange to lung protection [5, 6]. In this context, more recently, an independent detrimental role of higher inspiratory flow on the development of VILI has been suggested by preclinical studies [7, 8]. If true, lowering inspiratory flow could become another target for lung-protective ventilation.

The lungs behave as viscoelastic bodies and require more pressure to be inflated at any given volume when inspiratory flow is high. This “additional pressure” employed during inspiration at high inspiratory flows might induce preferential ventilation of lung units with short time constants, which will receive tidal volume first [9]. “Classical” consequences of such potentially harmful additional pressure and uneven distribution of ventilation are stress relaxation and pendelluft: when inspiratory flow ends and volume is held constant during an end-inspiratory pause, the pressure decreases releasing part of the accumulated parenchymal tension (stress relaxation) and gas redistributes to units with longer time constants (pendelluft). Of note, higher values of the dynamic additional pressure due to a higher flow rate have been associated with VILI development in the preclinical setting [8].

Electrical impedance tomography (EIT) is a recently introduced technique which relies on regional changes in thoracic impedance to small electrical currents applied to the skin to measure dynamic gas distribution inside the lungs. EIT is increasingly used in ARDS patients to dynamically measure ventilation heterogeneity [10, 11]; thus, we reasoned that EIT could further characterize the effects of high inspiratory flow on ventilation maldistribution.

The application of higher positive end-expiratory pressure (PEEP) could potentially mitigate higher flow effects by recruiting lung units and making ventilation more homogeneous [12], but it could also worsen them by overdistending already open alveoli [13].

The aim of our study was to explore the effects of increasing inspiratory flow rates keeping a constant tidal volume on the additional inspiratory pressure, pendelluft, and ventilation heterogeneity at lower vs higher PEEP. Our hypothesis was that increasing flow rate would result in worse distribution of ventilation and that higher PEEP levels could limit this phenomenon.

Some of the results of this study have been previously reported in the form of an abstract [14].

## Methods

Additional methods can be found in Additional file 1.

### Study population

We conducted a prospective randomized crossover study on ten intubated patients with ARDS, deeply sedated and paralyzed as per clinical decision, admitted to the general intensive care unit (ICU) of the Fondazione IRCCS Ca’ Granda Ospedale Maggiore Policlinico, Milan, Italy. Exclusion criteria included age <18 years; pregnancy; hemodynamic instability; pneumothorax; history of severe chronic obstructive pulmonary disease (COPD); history of nasal trauma and/or deviated nasal septum; contraindication to EIT use (e.g., presence of a pacemaker or an automatic implantable cardioverter defibrillator); impossibility to position the EIT belt (e.g., presence of wound dressings or chest drains); impossibility to position the esophageal pressure catheter (e.g., esophageal surgery); PaO_2_/FiO_2_ ≤ 100 mmHg; and clinically selected PEEP ≥15 cmH_2_O. The institutional ethical committee approved the study (reference number 364_2017), and informed consent was obtained according to local regulations. Demographic data, ARDS etiology, and severity at enrollment and ICU mortality were recorded.

### Advanced respiratory monitoring

An esophageal balloon catheter (CooperSurgical, Trumbull, CT) was advanced through the nose and its position checked by a standard method [15].

Flow, airway (Paw), and esophageal (Pes) pressures were continuously recorded and processed on a dedicated data acquisition system (Colligo; Elekton, Milan, Italy).

### EIT monitoring

The EIT belt was placed around the patient’s chest at the fifth or sixth intercostal space and connected to a commercial EIT monitor (PulmoVista 500; Dräger Medical GmbH, Lübeck, Germany). EIT data were registered at 20 Hz and stored for offline analysis, as previously described [16].

### Study protocol

Patients were ventilated in volume control mode (S1, Hamilton Medical, Bonaduz, Switzerland) with a tidal volume (Vt) of 6 ml/kg of predicted body weight, no end-inspiratory pause, clinical PEEP, FiO_2_ to obtain a SpO_2_ between 90 and 96%, and respiratory rate set to obtain an arterial pH of 7.30–7.45. Patients were kept supine and the backrest of the bed was positioned at an angle ≥30°.

The study consisted of two randomized crossover steps:

- PEEP 5 cmH_2_O
- PEEP 15 cmH_2_O

During each PEEP step, the inspiration to expiration ratio (I:E) was sequentially modified to obtain an inspiratory airflow of 400, 800, or 1200 ml/s (low, medium, or high inspiratory flow, respectively). Each flow set lasted for 10 min, and at the end of each step, end-inspiratory and end-expiratory holds were performed. At the start of each PEEP step, a recruitment maneuver was performed (1 min of pressure-controlled ventilation with a respiratory rate of 10, I:E of 1:1, end-inspiratory airway pressure of 40 cmH_2_O, and PEEP of 5 or 15) to normalize lung history. A timeline of the study protocol and the measurements performed is reported in the Methods section of Additional file 1.

### Offline airway, esophageal pressure, and EIT data analysis

**Fig. 1:**
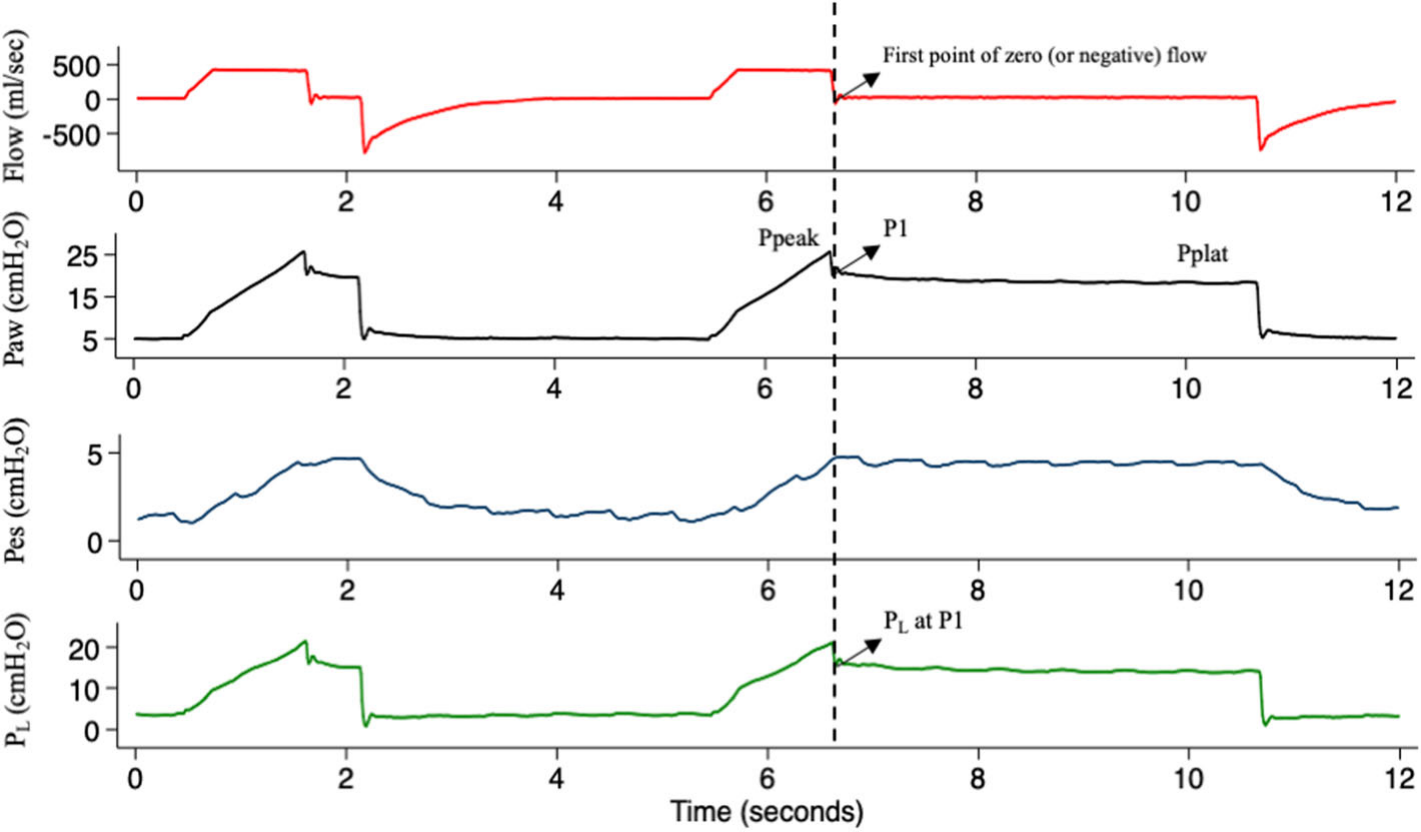
Method for P1 calculation. Flow–time trace (red) and airway (Paw, black), esophageal (Pes, blue), and transpulmonary (P_L_, green) pressure–time traces of a representative patient during an end-inspiratory occlusion. Transpulmonary pressure trace is obtained by subtraction of esophageal pressure trace from airway pressure trace. Peak pressure (Ppeak) is the highest (airway or transpulmonary) pressure value reached during inspiration. P1 is calculated as the point on the (airway or transpulmonary) pressure–time trace corresponding to the first zero or negative flow value on the flow–time trace after end-inspiratory occlusion. Plateau pressure (Pplat) is calculated as the (airway or transpulmonary) pressure value after 3 s from end-inspiratory occlusion

Peak (Ppeak) and plateau (Pplat) airway pressures and total PEEP (PEEPtot) were measured after end-inspiratory and end-expiratory holds. The pressure corresponding to the first point of zero (or negative) end-inspiratory flow on the airway pressure–time curve was defined as P1 (Fig. 1). Driving pressure was calculated as Pplat − PEEPtot and additional inspiratory pressure as P1 − Pplat.

**Fig. 2:**
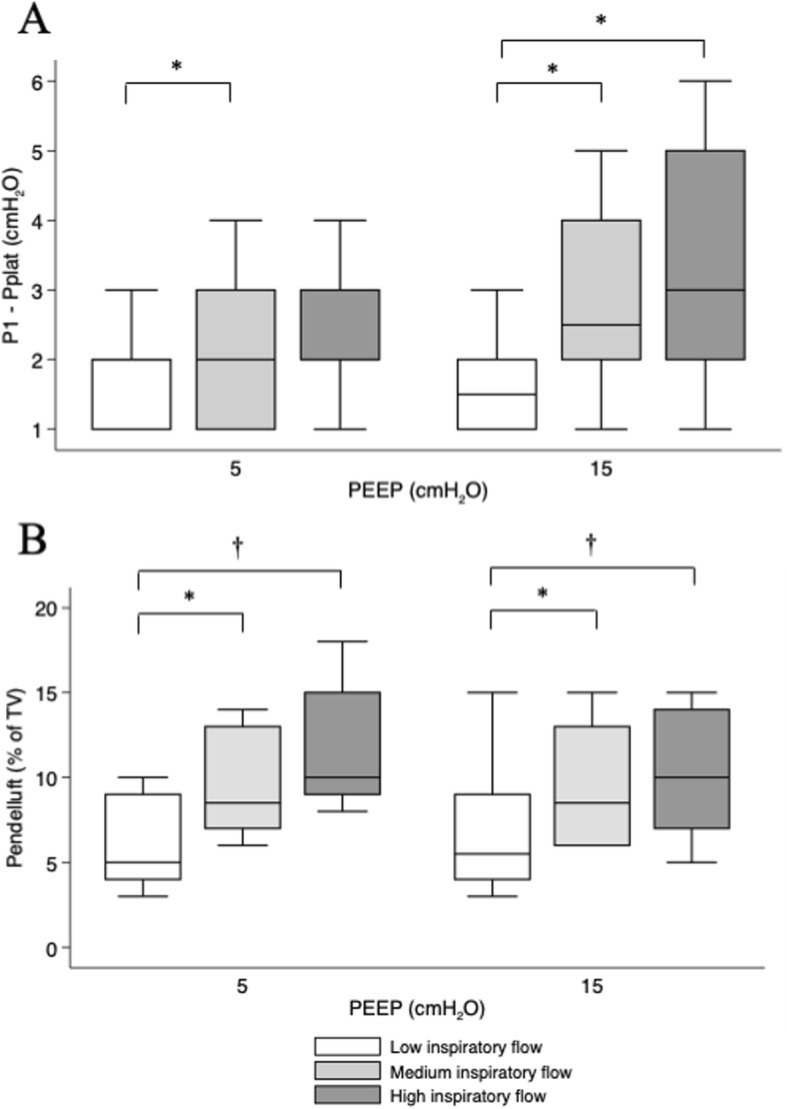
Flow and PEEP effect on additional inspiratory pressure and pendelluft. Box plot of additional flow-dependent pressure (measured as P1 − Pplat, **a**) and pendelluft (**b**) at the different combinations of PEEP (5 and 15 cmH_2_O) and inspiratory flow (400, 800, and 1200 ml/s; white, light gray, and dark gray, respectively). **a** *p* < 0.001 for flow effect, *p* = 0.168 for PEEP effect; **b** *p* < 0.001 for flow effect, *p* = 0.676 for PEEP effect; two-way repeated-measures ANOVA. *p < 0.05 within PEEP; †*p* < 0.01 within PEEP

Absolute (P_L_) and delta (ΔP_L_) transpulmonary pressures were computed from airway and esophageal pressure traces.

From raw EIT data, the following parameters were recorded:

- Homogeneity of the antero-posterior distribution of tidal volume (Vt_ndep_/Vt_dep_) [17]
- Global inhomogeneity (GI) index [18]
- Pendelluft: two EIT images, one corresponding to P1 and the other to Pplat, were recorded for each PEEP and flow step. The sum of the absolute values of the pixel-by-pixel difference between the two masks was used to quantify pendelluft.

### Statistical analysis

Given the exploratory and physiological nature of this study, we chose a reasonable sample size based also on the authors’ previous experience [17]. Normal distribution was tested by the Shapiro–Wilk test. Data are presented as the mean ± standard deviation (SD) or median and interquartile range (IQR) for continuous variables, as appropriate. Absolute or relative frequencies are used for categorical variables. Correlations between continuous variables were tested using linear regression analysis.

The study main objective was to describe the effect of inspiratory flow on additional inspiratory pressure, heterogeneity of ventilation distribution, and pendelluft at two different PEEP levels. Differences between these variables across inspiratory flow rates obtained during each PEEP step were tested by repeated-measures two-way analysis of variance (RM-ANOVA) on ranks with post-hoc Bonferroni correction. A level of *p* < 0.05 (two-tailed) was considered as statistically significant. Statistical analyses were performed with SigmaPlot 12.0 (Systat Software, Inc., San Jose, CA, USA).

## Results

### Patients’ characteristics

Patients’ characteristics are listed in Table 1. Patients were 64 ± 14 years old and 4 (40%) were women. The etiology of respiratory failure was infectious in 7 patients and primary in 4 patients. All patients were enrolled within 7 days from intubation. Five patients had mild and 5 had moderate ARDS at enrollment. Clinically selected PEEP ranged between 8 and 14 cmH_2_O and 4 patients died during their ICU stay.

**Table 1:** Patients’ clinical characteristics

Patient	Age	Gender	BMI	Risk factor for ARDS	SOFA at enrollment	SAPSII at ICU admission	Clinical PEEP	PaO_2_/FiO_2_ at clinical PEEP	ICU outcome
1	61	Female	26.3	Sepsis	5	32	12	190	Survivor
2	32	Male	24.2	Aspiration	11	70	8	187	Nonsurvivor
3	52	Female	23.9	Aspiration	6	50	8	235	Survivor
4	64	Female	36.7	Aspiration	10	77	12	246	Survivor
5	72	Female	21.2	Septic shock	11	81	10	170	Survivor
6	64	Male	27.7	Bacterial pneumonia	9	54	14	244	Survivor
7	73	Male	29.3	Septic shock	9	57	8	190	Nonsurvivor
8	80	Male	26.1	Septic shock	10	54	14	117	Survivor
9	59	Male	20.8	Septic shock	13	73	10	207	Nonsurvivor
10	81	Male	28.1	Septic shock	11	69	14	225	Nonsurvivor
Mean ± SD	64 ± 14	5 males/5 females	26.4 ± 4.6		10 ± 2	62 ± 15	11 ± 3	201 ± 40	6 survivors/4 nonsurvivors

### Respiratory mechanics at increasing flow and PEEP

The measured inspiratory flow during the study was 397 ± 37 ml/s, 838 ± 60 ml/s, and 1240 ± 105 ml/s (average of both PEEP levels) for low, medium, and high inspiratory flow steps, respectively. Plateau airway pressure did not change with increasing inspiratory flow and driving airway pressure was always below 14 cmH_2_O during the study. Peak airway pressure and P1 increased with both increasing PEEP and increasing inspiratory flow (Table 2).

**Table 2:** Classical respiratory mechanics parameters

	PEEP 5 cmH_2_O			PEEP 15 cmH_2_O			*p* value		
	Inspiratory flow			Inspiratory flow			Flow	PEEP	Interaction
	Low	Medium	High	Low	Medium	High			
Ppeak, cmH_2_O	21^*,†^ [19–24]	28^†^ [23–33]	39 [34–45]	31^*,†^ [28–33]	36^†^ [35–41]	47 [44–51]	<0.001	<0.001	<0.001
P1, cmH_2_O	17^†^ [15–20]	17 [16–19]	18 [17–20]	27 [25–29]	28 [26–29]	28 [26–31]	0.007	<0.001	0.889
Pplat, cmH_2_O	15 [15–18]	15 [14–16]	15 [14–16]	26 [24–26]	25 [23–26]	26 [24–27]	0.262	<0.001	0.568
PEEPtot, cmH_2_O	6^†^ [5–7]	6 [5–6]	5 [5–6]	16^*,†^ [15,16]	15 [15–15]	15 [15–15]	0.003	<0.001	0.442
Driving P, cmH_2_O	10 [8–11]	9 [8–11]	10 [8–11]	10 [9–11]	10 [8–11]	10 [9–12]	0.017	0.539	0.455

*Ppeak* peak airway pressure, *P1* end-inspiratory airway pressure at 0 flow, *Pplat* end-inspiratory plateau airway pressure, *PEEPtot* total (set + intrinsic) positive end-expiratory pressure, *Driving P* driving airway pressure

*p < 0.05 vs medium inspiratory flow; †*p* < 0.05 vs high inspiratory flow

End-expiratory absolute transpulmonary pressure, measured as the difference between airway and esophageal pressures, was negative at low PEEP and slightly positive at high PEEP. Absolute transpulmonary pressure at P1 increased with increasing flow and PEEP, while absolute transpulmonary pressure at Pplat changed only with PEEP (Table 3). Similar results, although with different absolute values, were found when measuring transpulmonary pressure as the difference between end-inspiratory (at either P1 or Pplat) and end-expiratory values (Table 3).

**Table 3:** Lung stress and heterogeneity parameters

	PEEP 5 cmH_2_O			PEEP 15 cmH_2_O			*p* value		
	Inspiratory flow			Inspiratory flow			Flow	PEEP	Interaction
	Low	Medium	High	Low	Medium	High			
P_L_ at P1, cmH_2_O	2 [0–4]	2 [1–3]	2 [1–4]	9^†^ [6–11]	8^†^ [7–11]	10 [9–12]	0.004	<0.001	0.209
P_L_ at Pplat, cmH_2_O	1 [0–2]	1 [0–2]	0 [−1–1]	6 [5–9]	8 [5–9]	9 [6–10]	0.304	<0.001	0.219
P_L_ at PEEPtot, cmH_2_O	−5 [−8–−3]	−5 [−7–−3]	−6 [−8–−3]	1 [1–2]	2 [1–2]	2 [1–3]	0.944	<0.001	0.011
ΔP_L_ at P1, cmH_2_O	7 [5–9]	7 [6–9]	9 [7–11]	6^†^ [4–9]	7 [6–8]	9 [6–10]	0.010	0.697	0.767
ΔP_L_ at Pplat, cmH_2_O	6 [4–7]	6 [5–7]	6 [5–9]	6 [3–7]	6 [5–6]	6 [5–6]	0.250	0.637	0.906
Vt_ndep_/Vt_dep_	1.69 [1.60–2.61]	1.72 [1.63–2.86]	1.73 [1.61–2.79]	1.37^*,†^ [1.20–1.47]	1.42 [1.23–1.56]	1.41 [1.23–1.54]	0.002	0.003	0.267
GI	82^†^ [67–88]	85 [68–91]	85 [68–92]	61 [58–66]	59 [58–67]	59 [57–67]	0.050	<0.001	0.148

*PL* absolute transpulmonary pressure, *P1* end-inspiratory airway pressure at 0 flow, *Pplat* end-inspiratory plateau airway pressure, *Pplat* end-inspiratory plateau airway pressure, *PEEPtot* total (set + intrinsic) positive end-expiratory pressure, *ΔP*_*L*_ delta transpulmonary pressure, *GI* global inhomogeneity index

*p < 0.05 vs medium inspiratory flow; †*p* < 0.05 vs high inspiratory flow

### Heterogeneity of ventilation

The difference between P1 and Pplat increased with increasing inspiratory flow at both PEEP levels (Fig. 2a). Ventilation redistribution at end-inspiration after the end of flow (i.e., the magnitude of pendelluft) increased at higher flows (Fig. 2b); PEEP did not prevent nor reduced pendelluft. A correlation was found between P1–Pplat and pendelluft at PEEP 15 cmH_2_O (*p* < 0.001, *R*^2^ 0.49), while no correlation was found at PEEP 5 cmH_2_O (*p* = 0.55) (Additional file 1: Figures S1 and S2).

EIT-derived indices of ventilation distribution showed an increase in heterogeneity of ventilation at higher inspiratory flows, while higher PEEP reduced both the global inhomogeneity index and gravitational heterogeneity of tidal volume distribution (Table 3). No correlation was found between P1−Pplat and both indices of heterogeneity of ventilation (Additional file 1: Figures S3–S6).

**Fig. 3:**
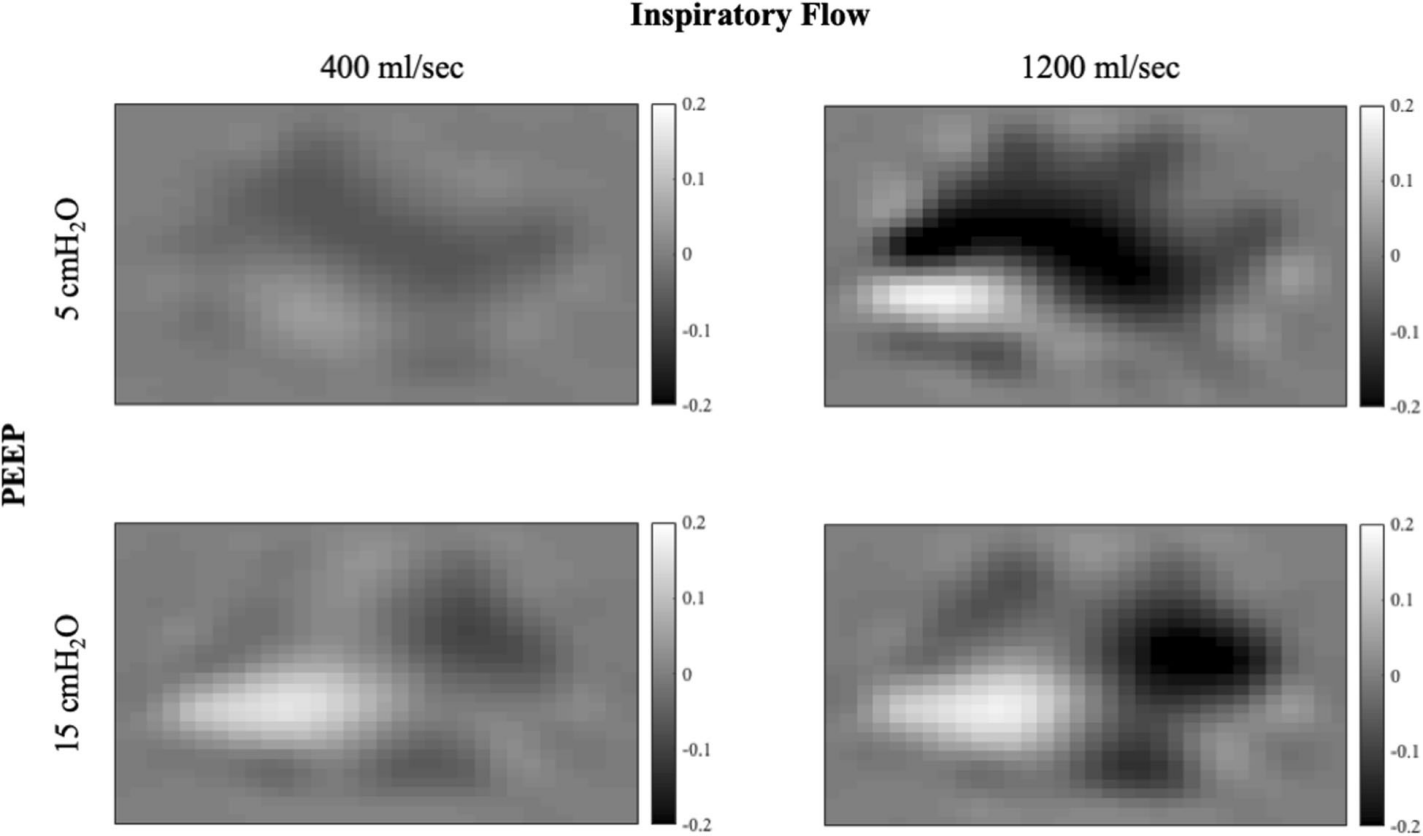
Pendelluft at end-inspiration. Pendelluft occurring at end-inspiration at PEEP 5 cmH_2_O (upper panels) and 15 cmH_2_O (lower panels) at low (left panels) and high (right panels) inspiratory flow in a representative patient. Pendelluft was calculated from the pixel-by-pixel difference between EIT-derived aeration at Pplat and P1 (see text for details). Each pixel was color-coded based on the amount of gas in milliliters entering (white) or leaving (black) the pixel. The color bar is provided on the right-hand side of each panel

A representative EIT image of pendelluft occurrence at low and high flows and both PEEP levels is shown in Fig. 3. Black pixels represent areas in which aeration decreases during the end-inspiratory pause, while white pixels represent areas in which aeration increases. Higher inspiratory flow at both PEEP levels is associated with a greater amount of gas movement within the lungs after the end of inspiration (pendelluft).

## Discussion

In this study, we sought to explore the relationship between higher inspiratory flow and additional risk of lung injury, as assessed by additional flow-related lung stress, end-inspiratory pendelluft, and regional ventilation imbalances and how PEEP might affect this relationship. We described higher levels of P1–Pplat at increasing flow rates at both low and high PEEP. EIT analysis showed a higher amount of pendelluft and less homogeneous distribution of tidal volume at higher airflows. While higher PEEP globally reduced heterogeneity of ventilation, it had no mitigating effect on pendelluft and maldistribution of tidal volume due to higher flows.

Our hypothesis was based on knowledge of lung viscoelastic properties and the pathophysiology of ARDS. The lungs, even healthy ones, are not perfectly elastic bodies. An elastic material elongates when subject to an external force, retains all the energy applied during elongation as potential energy, and uses it to come back to its original shape when the external force is withheld. A viscoelastic material instead needs more energy to be elongated by an external force than the one needed to come back to its original shape. The amount of energy applied to the body, but not used, is lost or “dissipated” to win internal resistance to elongation (i.e., molecular bonds and fibril entanglements in the lung fibrous skeleton) [19]. A way to unmask this behavior is to keep the elongation constant (e.g., during an end-inspiratory pause) and measure the decay over time of the internal stress produced inside the body (stress relaxation). When the respiratory system is subject to an end-inspiratory pause, pressure first decays rapidly from Ppeak to a lower value (P1, representing alveolar pressure), while flow decreases to zero. This first, rapid pressure drop is due to airway (both artificial and anatomic) resistance to flow. Afterwards, when inspiratory flow ceases, a slower pressure drop is observed. This additional pressure dissipation is due to air redistribution between alveoli (pendelluft) and parenchymal stress relaxation and can be measured as a decay on the airway or transpulmonary pressure–time curve from immediately after flow interruption (P1) to a plateau value (Pplat) (Fig. 1). During dynamic conditions, P1 reflects alveolar pressure more closely than Pplat and could thus be used to make inferences regarding the real pressures acting on the lung parenchyma. Of note, higher P1–Pplat values can indicate a less homogeneous distribution of tidal volume [9] and have been associated to lung damage in the preclinical setting [8].

But why should higher inspiratory flow increase the maldistribution of ventilation and (possibly) damage? ARDS is a heterogeneous disease. Open lung units coexist with alveoli filled with edema or partially collapsed under the weight of the overhead parenchyma [20]. The degree of aeration and small-airway plugging by edema and secretions is extremely variable; thus, wide differences in local compliance and resistance can coexist within the lung [12, 21]. Lung units with different time constants (i.e., compliance times resistance) inflate at different rates: long time constant units are “slow” while short time constant units are “fast” reacting. If airflow is slow enough, then the “slow” units will have time to be at (or almost at) equilibrium with airway opening pressure at the end of inspiration. With higher airflows instead, inspiratory time will not be enough for the “slow” units to equilibrate, while the “fast” unit will accommodate a greater fraction of the tidal volume [22]. During an end-inspiratory pause, “fast” units will partially deflate into the “slow” ones (pendelluft), and airway pressure will decrease accordingly (stress relaxation) [9]. This results in an increasingly uneven distribution of tidal volume with increasing inspiratory flow, possibly overdistending those alveoli receiving tidal volume first (“fast” units) and, in the long term, possibly promoting lung injury in those regions.

Although exploratory and in a small group of not very severe patients, our observations seem to partially confirm this framework: the higher the inspiratory flow in our patients, the higher the additional stress, pendelluft, and heterogeneity of tidal ventilation measured by EIT. We used two different indices to measure ventilation distribution. The global inhomogeneity index is a measure of global scatter in spatial distribution of impedance change (i.e., ventilation) within the lung during a tidal breath. The higher the global inhomogeneity index, the wider the degree of ventilation inhomogeneity between different lung units [18]. The second is Vt_ndep_/Vt_dep_, which measures the homogeneity of ventilation dependent on gravitational forces. This index equals 1 when the degree of ventilation of non-dependent and dependent units is similar, while values greater than 1 indicate preferential ventilation to non-dependent units [17]. As expected in mechanically ventilated, paralyzed, supine ARDS patients, this index was always >1 and higher PEEP reduced it, probably by recruiting dependent, previously closed, lung units. Recruitment can also be inferred by the change from negative to positive absolute transpulmonary pressure values at higher PEEP [23]. Despite recruitment and positive transpulmonary pressure at end-expiration at PEEP 15 cmH_2_O, high inspiratory flow acted to increase the proportion of tidal volume reaching non-dependent areas (Table 3), as also reported previously in healthy, upright subjects [24]. Both measures showed a slight but significant increase at higher airflows at both PEEP levels, while higher PEEP had a stronger effect in reducing inhomogeneity, irrespective of the applied inspiratory flow (Table 3). However, the changes in heterogeneity of ventilation due to high inspiratory flow were overall small in magnitude, and we cannot be sure of their clinical impact.

The intrinsic dynamic nature of EIT monitoring allows to visualize fast-occurring phenomena, such as pendelluft. In Fig. 3 the change in thoracic impedance during an end-inspiratory pause, i.e., not due to airflow from the airway opening, is represented. Since values in each pixel can assume both negative and positive values, the magnitude of pendelluft is the sum of the absolute values of each pixel. Interestingly, pendelluft showed a clear increase with higher inspiratory flow at both PEEP levels, while it was not affected by higher PEEP.

An unexpected finding of our study was the different effect that high PEEP had on P1–Pplat and EIT-derived heterogeneity of ventilation measures. In fact, while PEEP globally reduced heterogeneity of ventilation, it did not affect P1–Pplat and pendelluft. A possible explanation for this discrepancy is that P1–Pplat might reflect different phenomena all contributing to the dynamic additional inspiratory pressure. In addition to pendelluft, tissue resistance is another determinant of P1–Pplat, as internal tensile stress generated by friction between molecules during elongation slowly decays when enough time is given for molecular rearrangement to occur [19]. Other authors have previously reported that viscoelastic phenomena are increased in ARDS patients compared to healthy subjects and that higher PEEP increases stress relaxation [13, 25]. In these reports, however, it was not possible to isolate the relative contribution of time constant inequalities and tissue resistance. In our patients, P1–Pplat increased with increasing airflow and was not affected by higher PEEP, in a similar way to pendelluft. Furthermore, at high PEEP, a positive correlation was found between P1–Pplat and pendelluft. It is thus possible that pendelluft was a major determinant of the dynamic additional pressure, in particular at high PEEP when the other indices of heterogeneity of ventilation were globally reduced, while GI and Vt_ndep_/Vt_dep_ were probably more affected by recruitment of dorsal atelectatic parenchyma.

Whatever the mechanism, these results generate the hypothesis that any (putative) beneficial effect of PEEP in making ventilation more homogeneous might not be enough to prevent the potential detrimental effects of high inspiratory flow, which could therefore be considered a potential independent determinant of lung injury. Also, these preliminary results generate the hypothesis that, as part of the benefits of low tidal volume ventilation might be offset by the increase in inspiratory flow due to the higher respiratory rate, inspiratory flow could be minimized as part of a lung-protective strategy (e.g., using the lowest possible respiratory rates and/or adding extracorporeal CO2 clearance).

### Limitations

Some limitations of this study are worth mentioning. First, the sample size is small and composed of a quite variable population of mild–moderate ARDS patients. We believe however that the complexity of the protocol and the high number of measurements performed on each patient make these results reproducible and meaningful. Second, we chose not to enroll severe ARDS patients, because of the risk associated with the PEEP 5 cmH_2_O phase. No conclusion on severe ARDS can thus be drawn from these data. Third, in order to change inspiratory flow, we decided to keep the respiratory rate constant and change I:E. While in clinical practice inspiratory flow is more often elevated due to a high respiratory rate, many expert centers ventilate ARDS patients using a fixed inspiratory flow of 1 l/s, close to our high flow group. Furthermore, we wanted to isolate the effect of inspiratory flow from other possible effects (e.g., higher mechanical power). Increasing the respiratory rate might have the additional effect of inducing intrinsic PEEP, which could by itself change the distribution of tidal volume. Fourth, we studied sedated, paralyzed ARDS patients. These findings might not apply to patients in assisted modes of ventilation, in whom higher inspiratory flow might be beneficial as it has been associated with a reduction in the work of breathing [26]. Fifth, we chose to study two arbitrarily set levels of PEEP: 5 and 15 cmH_2_O. While we are aware that these two values might be associated with different levels of recruitment and overdistention in each patient, our choice was based on clinical practice, where 5 is a reasonably “low” PEEP and 15 a reasonably “high” PEEP for a mild–moderate ARDS patient. Lastly, EIT is influenced by changes in intrathoracic blood content, which in turn are affected by the ventilation modality employed, especially PEEP. The reduction of intrathoracic blood content expected to occur at higher PEEP might have reduced basal impedance at PEEP 15 cmH_2_O and thus complicate the comparison between the two PEEP levels. However, this impedance change is small compared to the much greater increase in impedance due to higher lung aeration. End-expiratory impedance change between the two PEEP levels was not a major objective of our study. Furthermore, the observed flow-dependent phenomena should be devoid of this “hemodynamic bias,” when they occur at the same PEEP level.

## Conclusions

Controlled ventilation with high inspiratory flow might increase lung additional stress and pendelluft in mild and moderate ARDS patients, while EIT-derived measures of ventilation heterogeneity are less affected. While increasing PEEP is effective in globally reducing the heterogeneity of tidal volume distribution, it might not reduce the maldistribution of ventilation and pendelluft caused by higher inspiratory flow. The absolute changes in heterogeneity and stress between flows were significant but small, and further studies are worth to describe the impact of these physiologic findings on the patient’s outcome.

## Supplementary information

**Additional file 1.** Additional methods and study results.

## Data Availability

The datasets used and/or analyzed during the current study are available from the corresponding author on reasonable request.

## Abbreviations

ARDS: Acute respiratory distress syndrome
EIT: Electrical impedance tomography
FiO2: Fraction of inspired oxygen
GI: Global inhomogeneity
ICU: Intensive care unit
P1: First point of zero end-inspiratory flow airway pressure
Paw: Airway pressure
PEEP: Positive end-expiratory pressure
PEEPtot: Total (set + intrinsic) PEEP
Pes: Esophageal pressure
P_L_: Absolute transpulmonary pressure
Ppeak: End-inspiratory peak airway pressure
Pplat: End-inspiratory plateau airway pressure
VILI: Ventilator-induced lung injury
Vt: Tidal volume
Vt_ndep_/Vt_dep_: Homogeneity of the non-dependent vs dependent distribution of ventilation
ΔP_L_: Delta transpulmonary pressure
