# Supplementary material for: Effects of inspiratory flow on lung stress, pendelluft, and ventilation heterogeneity in ARDS: a physiological study
Source: Crit Care. 2019 Nov 21;23:369. doi: 10.1186/s13054-019-2641-0 (PMC6873770; doi:10.1186/s13054-019-2641-0)
Supplement: Supplementary file 1 — Additional file 1. Additional methods and study results. [file 13054_2019_2641_MOESM1_ESM.docx]

**Effects of inspiratory flow on lung stress, pendelluft and ventilation heterogeneity in ARDS: a physiological study**

**ON-LINE DATA SUPPLEMENT**

Alessandro Santini^1,2^, Tommaso Mauri^1,3^, Francesca Dalla Corte^4^, Elena Spinelli^1^, Antonio Pesenti^1,3^

1 Department of Anesthesia, Critical Care and Emergency, Fondazione IRCCS Ca’ Granda Ospedale Maggiore Policlinico, Milan, Italy

2 Deparment of Anesthesia and Critical Care Medicine, Humanitas Clinical and Research Center, Rozzano (MI), Italy

3 Department of Pathophysiology and Transplantation, University of Milan, Milan, Italy

4 Department of morphology, surgery and experimental medicine, Azienda Ospedaliera-Universitaria Arcispedale Sant'Anna, University of Ferrara, Ferrara, Italy

**Additional Methods**

*Study protocol timeline*

*
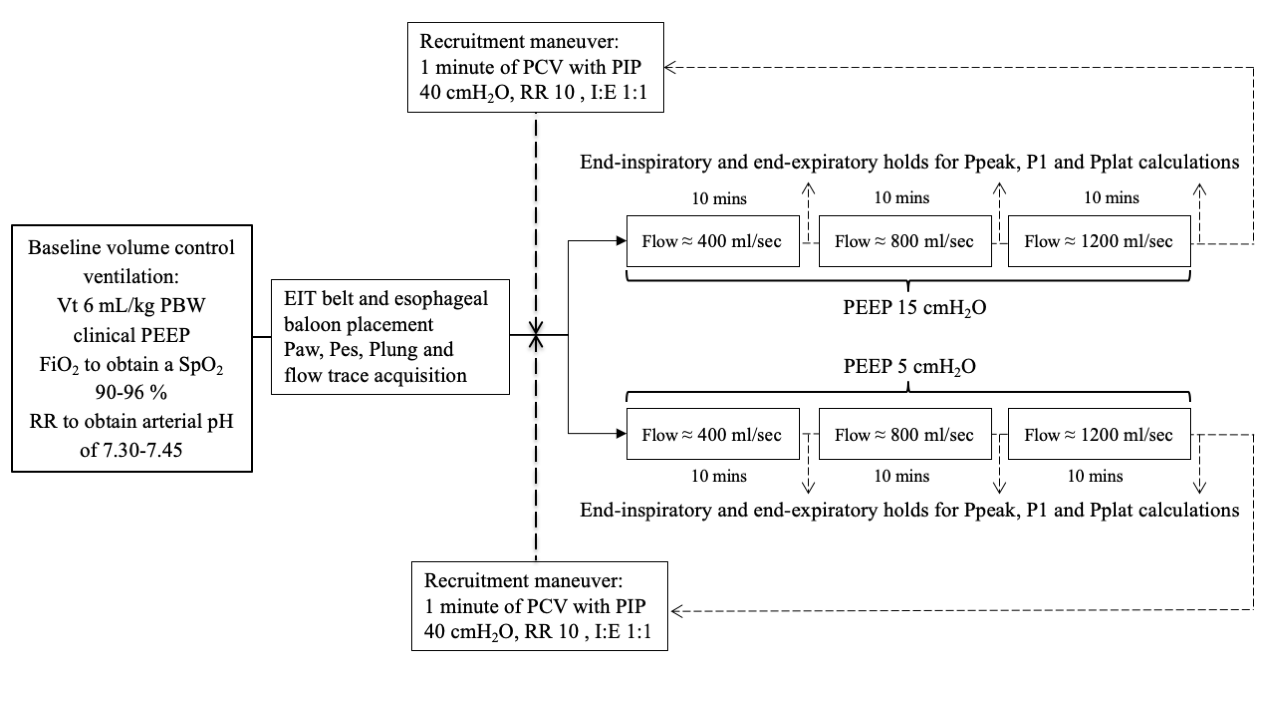
*

After baseline measurements, patient preparation and lung recruitment, randomization to PEEP 5 cmH_2_O or 15 cmH_2_O step occurred. At the end of the PEEP step a second recruitment maneuver was performed. Afterwards the remaining PEEP step was completed. Each PEEP step lasted approximately 45 minutes (10 minutes per flow step plus the time required to perform end-inspiratory and end-expiratory pauses), while the overall duration of the protocol (consisting of two crossover PEEP steps) was approximately 2 hours.

*Airway and lung mechanics*

Offline analyses of airway and esophageal traces recorded over a 1 minute period at the end of each flow and PEEP phase were performed.

Peak airway pressure (Ppeak), airway pressure at zero inspiratory flow following the end-inspiratory occlusion (P1), plateau pressure measured after 3 seconds from end-inspiratory occlusion (Pplat) and total PEEP (PEEPtot) were calculated after end-inspiratory and end-expiratory holds. Driving pressure was calculated as Pplat-PEEPtot. The additional inspiratory pressure was calculated as the difference between P1 and Pplat. Additional inspiratory pressure is the result of stress relaxation (i.e. tissue viscoelastic resistance) and pendelluft (i.e. time constant inequalities with gas redistribution at end-inspiration).

Absolute transpulmonary pressure (P_L_) was computed as the difference between Paw (either Ppeak, P1 or Pplat) and Pes (either Pes at Ppeak, Pes at P1 or Pes at Pplat).

Delta transpulmonary pressure (ΔP_L_), i.e. transpulmonary pressure above PEEP, was calculated as ΔPaw – ΔPes. ΔPaw is the difference between end-inspiratory (either P1 or Pplat) and end-expiratory Paw. ΔPes is the difference between end-inspiratory (either Pes at P1 or Pes at Pplat) and end-expiratory Pes.

*EIT-derived indices of heterogeneity of ventilation*

- Antero-posterior distribution of tidal volume (Vt_ndep_/Vt_dep_): we calculated a regional index of ventilation heterogeneity as the ratio between the number of ventilated pixel (i.e. pixels showing impedance changes < 10% of the maximal impedance change) in the non-dependent lung region and the number of ventilated pixel in the dependent regions. Values close to 1 indicate more homogenous distribution of tidal ventilation, values higher than 1 indicate more heterogeneous, preferentially non-dependent distribution of tidal ventilation
- Global Inhomogeneity Index (GI): the median impedance change of the pixels in the lung area was calculated during tidal breathing at each flow and PEEP level. GI was calculated as the sum of the absolute difference between the median value and every pixel value of the impedance change between inspiration and expiration, normalized to the sum of the impedance values within the lung area.
- Pendelluft: two EIT images (1024 pixels) for each PEEP and flow step were recorded at end-inspiration and analyzed offline. The first image corresponded to gas distribution at zero inspiratory flow (P1), the second image, corresponding to Pplat, was recorded 3 seconds after the first one. The P1-mask was subtracted pixel-by-pixel from the Pplat one, such that any positive value would mean an increase and any negative value a decrease, respectively, in gas content in that area during the pause. To quantify Pendelluft in millilters, the sum of the absolute values of impedance change in each pixel was multiplied by the tidal volume to absolute EIT units ratio measured in the previous tidal breath. Pendelluft was then expressed as percent of the delivered tidal volume.

**Additional Results**

*Figure E1 – P1-Pplat and pendelluft at PEEP 5 cmH_2_O*


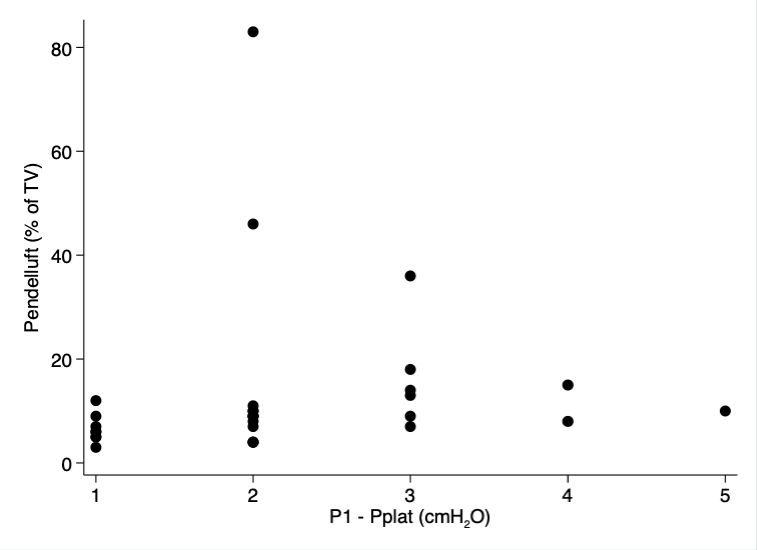


p = 0.55, R^2^ 0.01

*Figure E2 – P1-Pplat and pendelluft at PEEP 15 cmH_2_O*


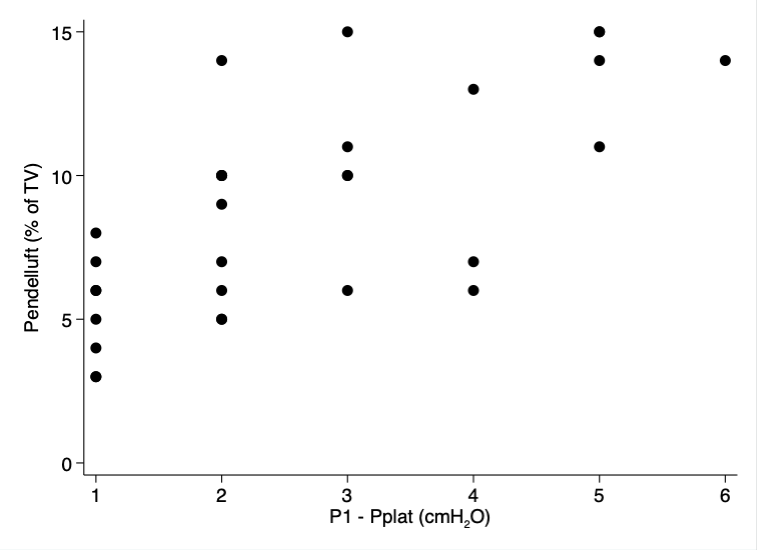


p < 0.001, R^2^ 0.49

*Figure E3 – P1-Pplat and global inhomogeneity index at PEEP 5 cmH_2_O*


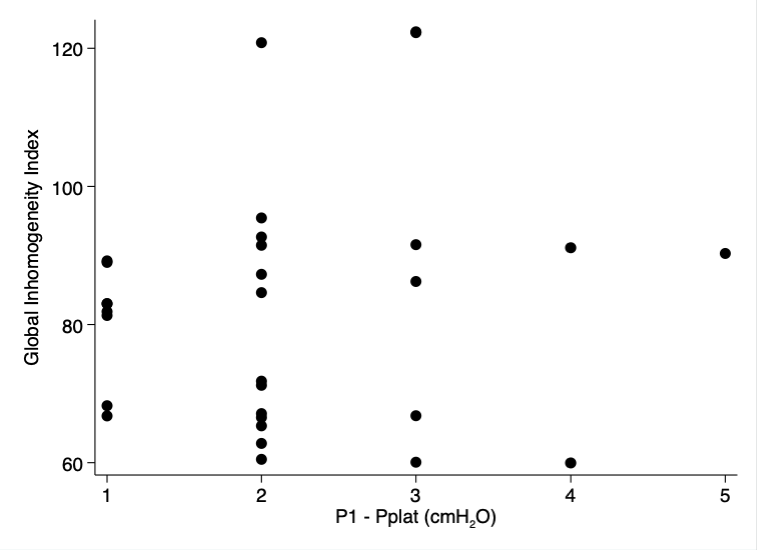


p = 0.49, R^2^ 0.02

*Figure E4 – P1-Pplat and Global Inhomogeneity Index at PEEP 15 cmH_2_O*


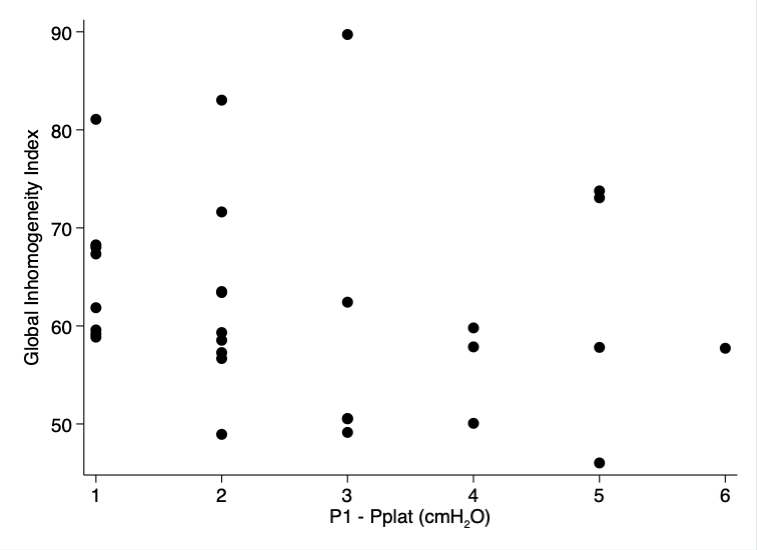
p = 0.32, R^2^ 0.03

*Figure E5 – P1-Pplat and Global Inhomogeneity Index at PEEP 5 cmH_2_O*


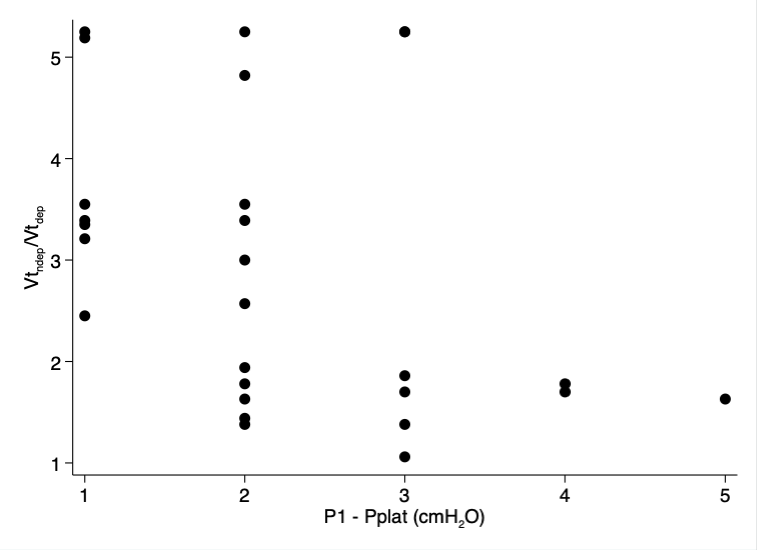


p = 0.33, R^2^ 0.03

*Figure E6 – P1-Pplat and Global Inhomogeneity Index at PEEP 15 cmH_2_O*


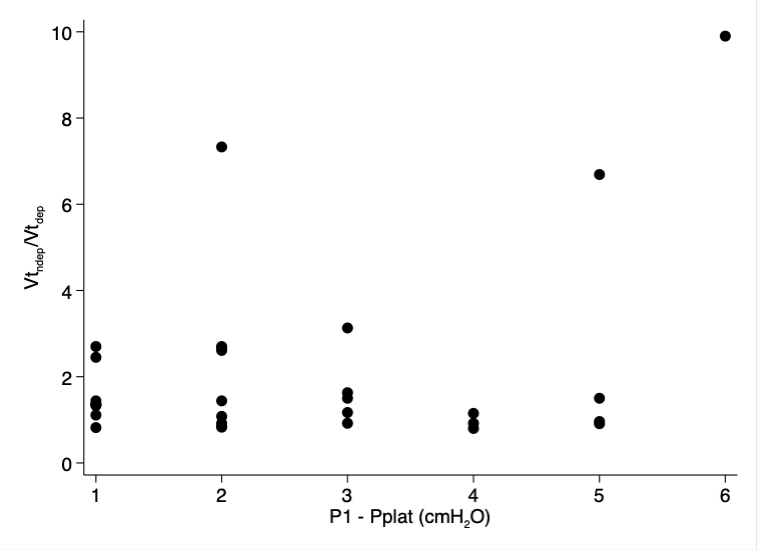


p = 0.07, R^2^ 0.10
